# Two decades of research on *Borrelia burgdorferi* sensu lato in questing *Ixodes ricinus* ticks in Slovakia

**DOI:** 10.3389/fcimb.2024.1496925

**Published:** 2024-12-13

**Authors:** Veronika Rusňáková Tarageľová, Markéta Derdáková, Diana Selyemová, Michal Chvostáč, Barbara Mangová, Yuliya M. Didyk, Juraj Koči, Stanislav Kolenčík, Bronislava Víchová, Branislav Peťko, Michal Stanko, Mária Kazimírová

**Affiliations:** 1 Institute of Zoology, Slovak Academy of Sciences, Bratislava, Slovakia; 2 Schmalhausen Institute of Zoology of the National Academy of Sciences of Ukraine, Kyiv, Ukraine; 3 Faculty of Natural Sciences, Comenius University in Bratislava, Bratislava, Slovakia; 4 Institute of Parasitology, Slovak Academy of Sciences, Košice, Slovakia; 5 Department of Epizootiology, Parasitology and Protection of One Health, University of Veterinary Medicine and Pharmacy, Košice, Slovakia

**Keywords:** *Borrelia*, prevalence, species diversity, habitat, long-term trends

## Abstract

**Introduction:**

In Europe*, Borrelia burgdorferi* sensu lato (s.l.), the causative agent of Lyme borreliosis is transmitted by the castor bean tick, *Ixodes ricinus*. In the last decades, global changes affect the spread of ticks and also their bionomics. The aim of this study was summarization of a large dataset obtained during 20 years of research.

**Methods:**

The research was carried out in 1999-2019 at 16 localities in Slovakia that were continuously monitored. In total, 17,249 questing *I. ricinus* ticks were tested for the presence of *B. burgdorferi* s.l.

**Results:**

The total prevalence of infected ticks was 18.8% (3,248/17,249), with 15.1% (1,557/10,302) infected nymphs and 24.3% (1,691/6,947) infected adults. Nine species of *B. burgdorferi* s.l. were identified. *Borrelia afzelii* (37.1%), *B. garinii*/*bavariensis* (24.7%), and *B. valaisiana* (15.4%) were the most frequent and were present at all study sites, followed by *B. lusitaniae* (12.6%), *B. burgdorferi* sensu stricto (4.1%) and *B. spielmanii* (1.6%). *Borrelia bavariensis* was confirmed only in four samples (0.1%), however, detection of this species has been performed only since 2017. *Borrelia bissettii* and *B. kurtenbachii* were both recorded in one case. The total prevalence differed significantly among four habitat types (urban, suburban, natural, agricultural). The highest infection prevalence was confirmed in natural habitat (22.0%), the lowest in urban habitat (13.2%). In addition, molecular analysis was carried out on part of the collected ticks previously morphologically identified as *I. ricinus*. The analysis did not confirm the occurrence of *Ixodes inopinatus* in Slovakia.

**Conclusion:**

Long-term monitoring of the abundance and spread of ticks as well as the prevalence and genetic variability of tick-borne pathogens can reveal the impact of global climatic and socio-economic changes on different habitats, including natural foci of tick-borne pathogens.

## 1 Introduction

Ticks transmit a wider range of pathogenic microorganisms than any other arthropod group (Durden, 2006). In humans, the diseases caused by these agents include Lyme borreliosis (LB), spotted fever group rickettsioses, human granulocytic anaplasmosis, tick-borne encephalitis, babesiosis, and others. Many of these diseases have emerged (or re-emerged) within the past decades (e.g. LB, anaplasmosis, rickettsioses, neoehrlichiosis). New foci of tick-borne disease (TBD) can be formed due to climatic changes and spread of ticks to new areas in the north and higher altitudes (Lindgren et al., 2000; Gray et al., 2009; Rusňáková Tarageľová et al., 2016).

*Ixodes ricinus* is the main vector of pathogenic microorganisms in Europe (Rizzoli et al., 2014), including *Borrelia* spirochaetes (Lindgren and Jaenson, 2006). Members of the *Borrelia burgdorferi* sensu lato (s.l.) complex are the causative agents of LB which is the most common TBD in areas of Eurasia with moderate climate (Stanek et al., 2012). This group comprises of more than 20 species transmitted by ticks from the *Ixodes ricinus* s.l. complex (Margos et al., 2019). *Borrelia afzelii*, *Borrelia garinii*, *Borrelia bavariensis*, *Borrelia spielmanii* and *Borrelia burgdorferi* sensu stricto (s.s.) are considered pathogenic for humans (Stanek et al., 2012). Genetic variability within the *B. burgdorferi* s.l. complex is associated with different clinical outcome in patients (van Dam et al., 1993) as well as with different reservoir hosts (Humair and Gern, 2000). Research on *B. burgdorferi* s.l. eco-epidemiology has a long history in Slovakia (rev. in Stanko et al., 2022). *Borrelia* prevalence in questing ticks was found to vary from 4.4% in northern Slovakia (Pangrácová et al., 2013) and up to 53.2% in eastern Slovakia (Venczel et al., 2016). The presence of nine species was confirmed, with *B. afzelii* and/or *B. garinii* as the most prevalent. Less frequent and rare species such as *Borrelia valaisiana, B. burgdorferi* sensu stricto (s.s.), *B. spielmanii, B. bavariensis, B. bissettii*, and *B. kurtenbachii* were reported as well (rev. in Stanko et al., 2022; Kazimírová et al., 2023). The prevalence of *Borrelia lusitaniae* was found to be low in Central Europe (Hubálek and Halouzka, 1997; Gern et al., 1999), nevertheless, natural foci with dominance of this species were confirmed in some areas of Slovakia (Rusňáková Tarageľová et al., 2016).

In order to find out whether there are changes in *Borrelia* prevalence and species distribution over the years, long-term monitoring of the prevalence, occurrence and species distribution is necessary. The main aim of this study is the evaluation of changes in the *B. burgdorferi* s.l. prevalence and diversity of species of the *B. burgdorferi* s.l. complex in selected sites of Slovakia during the period from 1999 to 2019.

*Ixodes inopinatus* described by Estrada-Peña et al. (2014) was confirmed in dry areas of the Mediterranean region in Spain, Portugal, Morocco, Algeria and Tunisia. This tick species morphologically resembles *I. ricinus*. Its occurrence has been confirmed also outside the Mediterranean region (Chitimia-Dobler et al., 2018) and it is possible that in the past many individuals of *I. inopinatus* were mistakenly classified as *I. ricinus*. However, the recent study of Rollins et al. (2023) calls into question the occurrence of *I. inopinatus* in Central Europe.

Hauck et al. (2019) confirmed the presence of *Borrelia* spp., *Rickettsia* spp. and *Anaplasma phagocytophilum* in *I. inopinatus*, while, compared to *I. ricinus*, a considerably higher prevalence of *Borrelia* was recorded in *I. inopinatus*. Molecular screening of selected ticks that were collected in Slovakia would clarify whether *I. inopinatus* also occurs there and participates, together with *I. ricinus*, in the maintenance and transmission of tick-borne pathogens.

## 2 Materials and methods

### 2.1 Tick sampling and study sites

**Figure 1:**
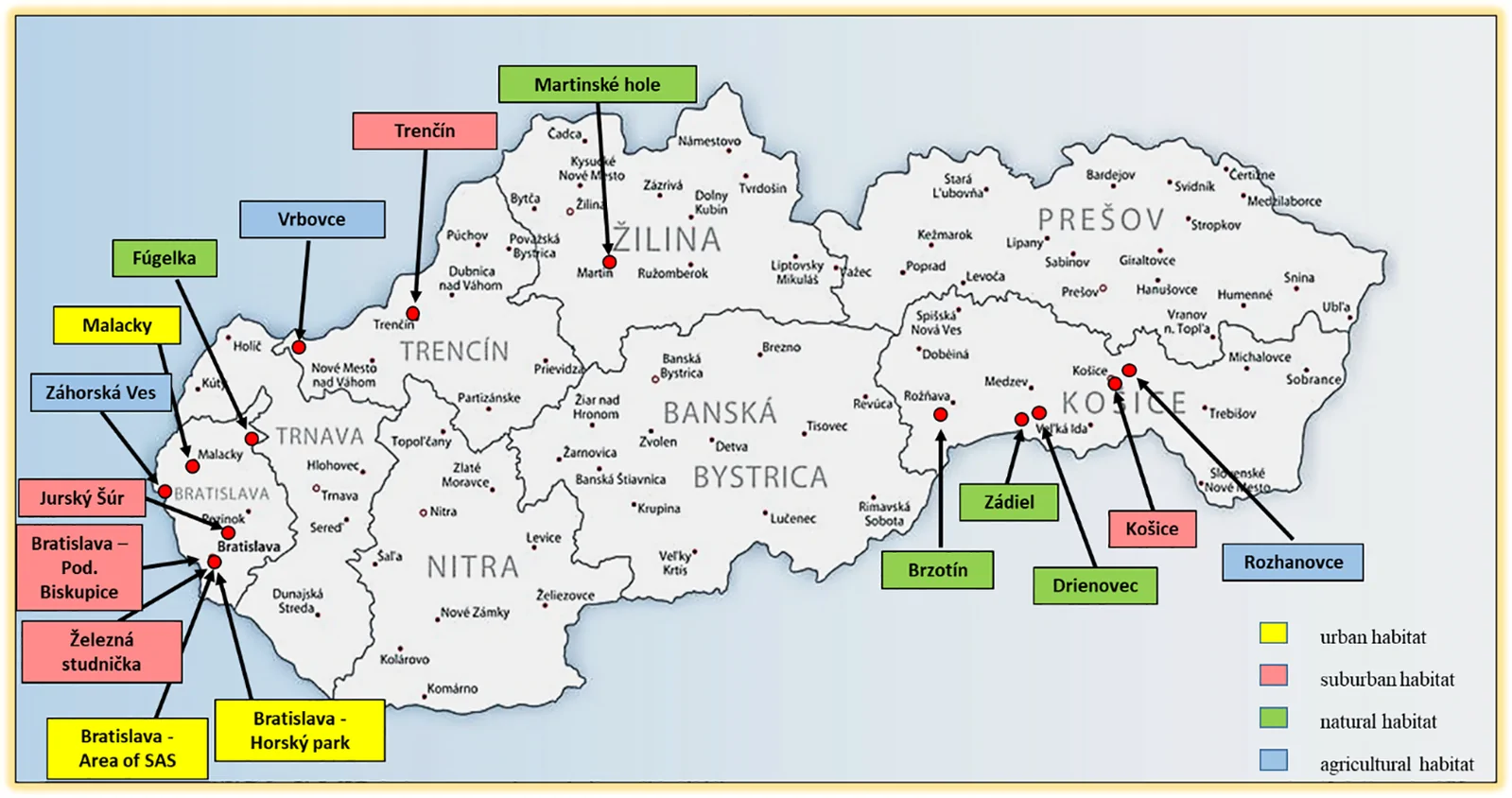
Study sites in Slovakia with the type of habitat.

Questing ticks were collected by flagging the vegetation at 16 study sites in Slovakia during 1999-2019 (**Figure 1**). Feeding ticks were collected from birds captured in Drienovec in 2019 (Šujanová et al., 2022). Chosen study locations represented different types of habitats: urban, suburban, natural and agricultural (**Supplementary Table S1**). The research was done in western Slovakia (WS): four study sites in Bratislava (the campus of the Slovak Academy of Sciences - SAS, Železná studnička, Horský park, Podunajské Biskupice), Malacky, Záhorská Ves, Jurský Šúr, Vrbovce, Fúgelka, Trenčín and in eastern Slovakia (ES): Košice, Rozhanovce, Zádiel, Drienovec, Brzotín. Study sites at Martinské hole Mountains represent a region of northern Slovakia (NS).

Urban habitat is represented by three sites: Horský park and the campus of the SAS in Bratislava, and the castle park in the centre of Malacky town. All these parks are fenced.

Suburban habitat is represented by five sites: Železná studnička and Podunajské Biskupice (both in Bratislava), Jurský Šúr nature reserve, Trenčín and Košice. These sites are not fenced and pass into open country or forest.

Natural habitat is represented by Fúgelka, Martinské hole, Drienovec, Zádiel and Brzotín.

Agricultural habitat is represented by Vrbovce, Záhorská Ves and Rozhanovce.

### 2.2 Identification of ticks

Questing and several bird-feeding ticks were morphologically identified to species, life stages and sex using standard keys (Nosek and Sixl, 1972; Slovák, 2010). To confirm the identification of ticks and find out whether *I. inopinatus* (Estrada-Peña et al., 2014) occurs in Slovakia, the mitochondrial 16S rRNA locus and nuclear gene TROSPA were amplified and sequenced. The sequences were compared via BLASTn (Altschul et al., 1990) searches to sequences available in GenBank (Mangold et al., 1998; Noureddine et al., 2011; Norte et al., 2021). To amplify each gene, published primers and protocols were used according to Mangold et al. (1998) for 16S rRNA and Noureddine et al. (2011) for TROSPA. Obtained sequences were compared with sequences for *I. ricinus* and *I. inopinatus* mentioned in the previous research (16S rRNA, GenBank PopSet: 309318023; TROSPA, GenBank PopSet; 309318631 and 309318389) (Noureddine et al., 2011; Poli et al., 2020).

### 2.3 Genomic DNA isolation and PCR detection of *B. burgdorferi* s.l.

DNA was isolated from individual ticks by using the alkaline hydrolysis method (Guy and Stanek, 1991) and/or by using commercial kits: DNeasy Blood & Tissue Kit (Qiagen, Germany) and/or Macherey-Nagel NucleoSpin^®^Tissue kit (Düren, Germany), following the manufacturer protocols. To confirm the presence of the tick DNA a 620-bp fragment of tick mitochondrial gene cytochrome *b* was amplified in randomly selected samples (Black and Roehrdanz, 1998) or total DNA was measured with a Nanodrop 2000c (Thermo Scientific, Wilmington, USA). Samples were stored at -20°C. Isolated DNA was screened for the presence of *B. burgdorgferi* s.l. by several PCR methods as described in previous publications (Derdáková et al., 2003; Hanincová et al., 2003a, 2003b; Taragel'ová et al., 2008; Chvostáč et al., 2018; Vaculová et al., 2019; Mtierová et al., 2020; Kazimírová et al., 2023). A positive (DNA from *Borrelia*-positive tick that was previously sequenced) and a negative control (Nuclease-Free Water, Qiagen, Germany instead of DNA) were used in each PCR reaction. The PCR products from the conventional PCRs were electrophoresed in a 1.5% agarose gel stained with GoodView™ Nucleic Acid stain (SBS Genetech, Beijing, China) and visualised with a UV transilluminator. The list of used PCR assays is summarised in **Supplementary Table S2**. *Borrelia*-positive samples were further genotyped by RLB (reverse line blot) (Rijpkema et al., 1995; Hanincová et al., 2003a, 2003b; Taragel'ová et al., 2008) or by RFLP (restriction fragment length polymorphism) (Derdáková et al., 2003; and/or sequencing (Chvostáč et al., 2018; Vaculová et al., 2019; Kazimírová et al., 2023). Selected positive samples were further analysed using MLST (multilocus sequence typing) according to the protocol by Margos et al. (2008) and (Mtierová et al., 2020). Samples positive for *B. garinii* have been sequenced for the identification of *B. bavariensis* prevalence only since 2017.

Temporal changes in the prevalence and genetic variability were evaluated only at study sites that had been monitored for at least five years.

### 2.4 PCR product purification, Sanger sequencing

Purification of PCR products for Sanger sequencing was done by NucleoSpin Gel and PCR Clean-up (Machery-Nagel, Düren, Germany). The sequencing in both the forward and reverse direction was performed in Eurofins Genomics (Eurofins Genomics Germany GmbH, Ebersberg, Germany) and by SeqMe (SEQme s.r.o., Dobříš, Czech Republic). The complementary strands of each sequenced product were manually assembled into consensus sequences. The sequences were compared with GenBank entries using the basic local alignment search tool algorithm (www.ncbi.nlm.nih.gov/blast) and aligned with representative homologous sequences using the Clustal W implemented in the MEGA software version 11 (Tamura et al., 2021).

### 2.5 Statistical analysis

Within the monitored years, the collections were not uniform, therefore we statistically evaluated only multi-year collections at the same sites (5 and more collection years). Statistical differences in *B. burgdorferi* s.l. prevalence between study years and between four different habitat types were evaluated by t-test using updated PAST 3 system package (Hammer et al., 2001). Chi-square test was used to analyse the proportions of collected nymphs and adult ticks and the prevalence levels for significant independence. The level of significance was set at p < 0.05. Ninety-five percent confidence intervals (95% CI) for each proportion were calculated using an online calculator (Sergeant, 2018) at the website https://epitools.ausvet.com.au/ciproportion. The program outputs include the estimated proportion plus upper and lower limits of the specified confidence interval, using Wilson Score interval method (Brown et al., 2001). Correlations between prevalence of *B. burgdorferi* s.l. species and habitat types were evaluated by Multivariate - Principal component analysis (PCA) using updated PAST 3 system package (Hammer et al., 2001).

## 3 Results

### 3.1 Questing ticks

In total, 17,249 *I. ricinus* ticks, including 10,302 nymphs and 6,947 adults were analysed from all study sites.

Questing adult ticks were significantly more abundant than nymphs at two sites in Bratislava: Podunajské Biskupice (χ^2^ = 30.70, p < 0.05), Železná studnička (χ^2^ = 31.42, p < 0.05) and in Záhorská Ves (χ^2^ = 13.11, p < 0.05) as well as at Martinské hole (χ^2^ = 27.82, p < 0.05) and in Košice (χ^2^ = 17.47, p < 0.05). On the contrary, nymphs were significantly more abundant at 10 sites: Vrbovce (χ^2^ = 30.92, p < 0.05), Malacky (χ^2^ = 230.12, p < 0.05), Šúr (χ^2^ = 6.45, p < 0.05), Fúgelka (χ^2^ = 432.77, p < 0.05), Bratislava – SAS (χ^2^ = 435.62, p < 0.05), Bratislava – Horský park (χ^2^ = 53.82, p < 0.05), Trenčín (χ^2^ = 32.11, p < 0.05), Rozhanovce (χ^2^ = 464.82, p < 0.05), Brzotín (χ^2^ = 12.46, p < 0.05), Drienovec (χ^2^ = 190.75, p < 0.05). At site Zádiel the nymphs/adults ratio was balanced (**Table 1**).

**Table 1:** Prevalence of *B. burgdorferi *s.l. in *Ixodes ricinus* ticks at study sites in Slovakia.

Type of habitat	Study site	No. of positive/tested adults (%)	95% CI	No. of positive/tested nymphs (%)	95% CI	No. of positive/tested total ticks (%)	95% CI
Urban	Bratislava – Horský park	23/110 (20.9)	14.4-29.4	32/249 (12.9)	9.3-17.6	55/359 (15.3)	12.0-19.4
	Bratislava – SAS	38/531 (7.2)	5.3-9.7	94/1463 (6.4)	6.3-7.8	132/1994 (6.7)	5.6-7.8
	Malacky	171/682 (25.1)	22.0-28.5	224/1369 (16.4)	14.5-18.4	395/2051 (19.3)	17.6-21.0
	Total	232/1323 (17.5)	15.6-19.7	350/3081 (11.4)	10.3-12.5	582/4404 (13.2)	12.3-14.3
Suburban	Bratislava – Železná studnička	217/1286 (16.9)	14.9-19.0	94/1017 (9.2)	7.6-11.2	311/2303 (13.5)	12.2-15.0
	Bratislava - Podunajské Biskupice	76/236 (32.2)	26.6-38.4	28/130 (21.5)	15.3-29.4	104/366 (28.4)	24.0-33.2
	Jurský Šúr	84/258 (32.6)	27.1-38.5	74/319 (23.2)	18.9-28.1	158/577 (27.4)	23.9-31.2
	Košice	239/892 (26.8)	24.0-29.8	123/724 (17.0)	14.4-19.9	362/1616 (22.4)	20.4-24.5
	Trenčín	12/43 (27.9)	16.8-42.7	28/114 (24.6)	17.6-33.2	40/157 (25.5)	19.3-32.8
	Total	628/2715 (23.1)	21.6-24.8	347/2304 (15.1)	13.7-16.6	975/5019 (19.4)	18.4-20.5
Natural	Fúgelka	83/632 (13.1)	10.7-16.0	263/1619 (16.2)	14.5-18.1	346/2251 (15.4)	13.9-16.9
	Martinské hole	390/1057 (36.9)	34.0-39.9	148/828 (17.9)	15.4-20.6	538/1885 (28.5)	26.6-30.6
	Drienovec	67/249 (26.9)	21.8-32.7	169/667 (25.3)	22.2-28.8	236/916 (25.8)	23.0-28.7
	Zádiel	21/86 (24.4)	16.6-34.5	10/88 (11.4)	6.3-19.7	31/174 (17.8)	12.8-24.2
	Brzotín	10/34 (29.4)	16.8-46.2	14/70 (20.0)	12.3-30.8	24/104 (23.1)	16.0-32.1
	Total	571/2058 (27.7)	25.6-29.7	604/3272 (18.5)	17.2-19.8	1175/5330 (22.1)	21.0-23.2
Agricultural	Záhorská Ves	208/456 (45.6)	41.1-50.2	66/353 (18.7)	15.0-23.1	274/809 (33.9)	30.7-37.2
	Vrbovce	9/109 (8.3)	4.4-15.0	50/208 (24.0)	18.7-30.3	59/317 (18.6)	14.7-23.3
	Rozhanovce	43/286 (15.0)	11.4-19.6	140/1084 (12.9)	11.1-15.0	183/1370 (13.4)	11.7-15.3
	Total	260/851 (30.6)	27.6-33.7	256/1645 (15.6)	13.9-17.4	516/2496 (20.7)	19.1-22.3
Total		1691/6947 (24.3)	23.4-25.4	1557/10302 (15.1)	14.4-15.8	3248/17249 (18.8)	18.3-19.4

### 3.2 Molecular identification of ticks

Randomly selected samples of tick DNA collected at Bratislava - Železná studnička, Malacky, Vrbovce, Martinské hole, Drienovec and Košice were sequenced (**Table 2**). Only samples with high quality sequences were included in the further analysis (n = 46). Sequenced samples from Drienovec (n = 16) represented ticks fed on birds: 3 ticks from *Turdus merula* (n = 2), 6 ticks from *Turdus philomelos* (n = 4), 3 ticks from *Erithacus rubecula* (n = 2), 1 tick from *Fringilla coelebs* (n = 1), 1 tick from *Coccothraustes coccothraustes* (n = 1), 1 tick from *Garrulus glandarius* (n = 1), 1 tick from *Parus major* (n = 1). Sequenced samples from other study sites were represented by ticks collected from vegetation. Morphological identification matched sequencing results, *I. ricinus* was confirmed in all tested samples. All our sequenced samples had 100% query coverage, E-value = 0, and 100% identity with more than one sample of *Ixodes ricinus* (KF197124.1 - Italy, KJ414457.1 – Belgium, GU074645.1 – France, OY973598.1) from the GenBank.

**Table 2:** List of *Ixodes* ticks molecularly identified to species (16S, TROSPA).

Study site	Year of ticks collection	Tick developmental stage/sex	No. of sequenced samples
Železná studnička	2017, 2018	5F	5
Malacky	2017, 2018	2N, 3F	5
Vrbovce	2017, 2018	2N, 1F, 2M	5
Martinské hole	2016	7F, 3M	10
Drienovec	2019	5L,11N**	16
Košice	2017, 2018	5N	5
Total		5L, 20N, 16F, 5M	46

L, larva; N, nymph; F, female; M, male; **bird-feeding ticks.

### 3.3 Prevalence of *B. burgdorferi* s.l.

A total of 17,249 *I. ricinus* ticks, including 10,302 nymphs and 6,947 adults were tested for the presence of *B. burgdorferi* s.l. The total prevalence of infected ticks was 18.8% (3248/17249) (95% CI: 18.3-19.4), with 15.1% (1557/10302) (95% CI: 14.4-15.8) infected nymphs and 24.3% (1691/6947) (95% CI: 23.4-25.4) infected adults. The differences in total prevalence among nymphs and adult ticks were not significant (χ^2^ = 2.15, p = 0.14). The lowest infection prevalence was detected at site Bratislava – SAS (6.6%, 95% CI: 5.6-7.8), the highest at site Záhorská Ves (33.9%, 95% CI: 30.7 – 37.2) (**Table 1**). Significantly higher infection prevalence in nymphs than adults was recorded at Vrbovce (χ^2^ = 7.63, p < 0.05). On the contrary, significantly higher infection prevalence in adult ticks than in nymphs was found at three sites: Záhorská Ves (χ^2^ = 11.25, p < 0.05), Martinské hole (χ^2^ = 6.59, p < 0.05) and Zádiel (χ^2^ = 4.72, p < 0.05). At the other sites no significant differences were recorded.

### 3.4 Species of *B. burgdorferi* s.l. identified in ticks

A total of 3,158 (97.2%) PCR-positive samples were successfully genotyped. Nine species of *B. burgdorferi* s.l. were identified (**Table 3**; **Supplementary Figure S1**). *Borrelia afzelii* (37.1%), *B. garinii*/*bavariensis* (24.7%), *B. valaisiana* (15.4%) were the most frequent and were present at all study sites. *Borrelia afzelii* was the dominant species at ten sites (Bratislava: Železná studnička, Horský park, SAS and Podunajské Biskupice, Záhorská Ves, Malacky, Vrbovce, Rozhanovce, Brzotín, Drienovská mokraď). The highest proportion of ticks harbouring this species was detected in Malacky (19.2%). *Borrelia garinii*/*bavariensis* was dominant at sites Fúgelka (18.7%) and Košice (18.0%) while *B. valaisiana* was the most frequent species at sites Záhorská Ves (13.0%) and Jurský Šúr (12.1%). *Borrelia burgdorferi* s.s. (4.1%) was found at majority of sites with the exception of Vrbovce, Jurský Šúr and Drienovská mokraď. This species was the most frequent in Košice (31.3%). *Borrelia spielmanii* (1.6%) appeared sporadically at three sites in Bratislava, in Malacky, Jurský Šúr, Fúgelka and at three sites in Eastern Slovakia with the dominance in Bratislava SAS and Malacky (25.5% each). *Borrelia lusitaniae* (12.6%) was recorded at all sites with the exception of Brzotín. This species considerably dominated in the Martinské hole area (73.7%). We confirmed the presence of *B. bavariensis* only in four ticks: 2 nymphs and 1 male from Malacky (0.1%) and one female from Fúgelka (0.03%).

**Table 3:** Number of positive *Ixodes ricinus* ticks and prevalence of *B. burgdorferi* s.l. species at study sites in Slovakia.

Species Study sites	*B. afzelii*	*B. garinii*/ *B. bavariensis*	*B. burgdorferi* s.s.	*B. valaisiana*	*B. lusitaniae*	*B. spielmanii*	*B. bavariensis*	mixed infections*	Total
BA-ŽS (%)	130 (43.9)	77 (26.0)	8 (2.7)	48 (16.2)	22 (7.4)	9 (3.0)	-	2 (0.7)	296
BA-HP (%)	18 (34.6)	17 (32.7)	4 (7.7)	10 (19.2)	1 (1.9)	2 (3.8)	-	0	52
BA-SAS (%)	40 (30.3)	32 (24.2)	10 (7.6)	23 (17.4)	13 (9.8)	13 (9.8)	-	1 (0.8)	132
BA-PB (%)	65 (62.5)	21 (20.2)	1 (1.0)	10 (9.6)	2 (1.9)	0	-	5 (4.8)	104
Záh. Ves (%)	128 (46.7)	30 (10.9)	8 (2.9)	63 (23.0)	25 (9.1)	0	-	20 (7.3)	274
Malacky (%)	225 (63.2)	64 (18.0)	10 (2.8)	38 (10.7)	3 (0.8)	13 (3.7)	3 (0.8)	19* (5.3)	375
Vrbovce (%)	29 (59.2)	8 (16.3)	0	6 (12.2)	2 (4.1)	0	-	4 (8.2)	49
Jurský Šúr (%)	13 (8.2)	66 (41.8)	0	59 (37.3)	1 (0.6)	1 (0.6)	-	18 (11.4)	158
Fúgelka (%)	120 (35.0)	146 (42.6)	9 (2.6)	44 (12.8)	14 (4.1)	2 (0.6)	1 (0.3)	7 (2.0)	343
Trenčín (%)	7 (17.5)	18 (45.0)	4 (10.0)	10 (25.0)	1 (2.5)	0	-	0	40
Mart. hole (%)	113 (21.5)	62 (11.8)	19 (3.6)	28 (5.3)	294 (55.9)	0	-	10 (1.9)	526
Košice (%)	83 (24.4)	140 (41.2)	41 (12.1)	50 (14.7)	1 (0.3)	1 (0.3)	-	24 (7.1)	340
Rozhanovce (%)	62 (36.3)	40 (23.4)	14 (8.2)	37 (21.6)	13 (7.6)	5 (2.9)	-	9** (3.5)	180
Zádiel (%)	4 (12.9)	2 (6.5)	2 (6.5)	14 (45.2)	2 (6.5)	5 (16.1)	-	2 (6.5)	31
Brzotín (%)	10 (47.6)	6 (28.6)	1 (4.8)	4 (19.0)	0	0	-		21
Drienovec (%)	126 (53.4)	49 (20.8)	0	42 (17.8)	5 (2.1)	0	-	14 (5.9)	236
Total (%)	1173	779	131	486	399	51	4	135	3158
Prevalence	37.1%	24.7%	4.1%	15.4%	12.6%	1.6%	0.1%	4.3%	

BA-PB, Bratislava - Podunajské Biskupice; BA-SAS, Bratislava – Slovak Academy of Sciences; BA-HP, Bratislava – Horský park; BA-ŽS, Bratislava – Železná studnička. *one tick infected with *B. bissettii*; **one tick infected with *B. kurtenbachii*.

**Figure 2:**
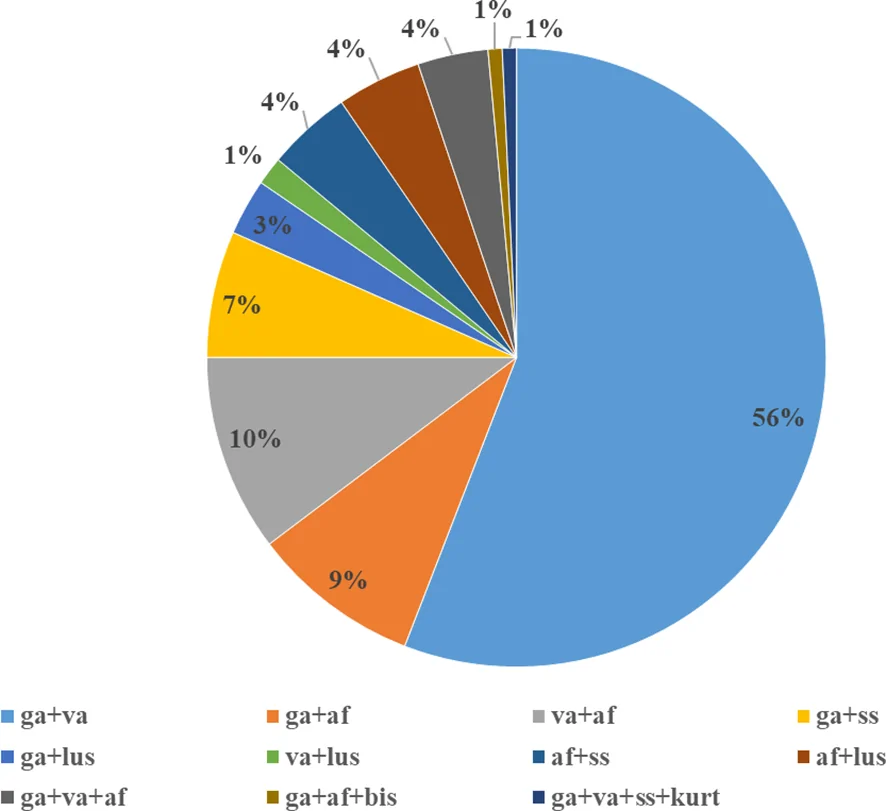
*Borrelia burgdorferi* s.l. species in mixed infections. ga - *B. garinii*, va - *B. valaisiana*, af - *B. afzelii*, ss - *B. burgdorferi* s.s., lus - *B. lusitaniae*, bis - *B. bissettii*, kurt - *B. kurtenbachii*.

Co-infections with two, three and/or four species were detected in 4.3% of infected ticks. Among mixed infections, *B. garinii* + *B. valaisiana* was the most frequent (56.3%) followed by *B. afzelii* mixed with *B. valaisiana* (10.4%) and *B. garinii* (8.9%). Prevalence of other species combinations ranged from 1.5 to 6.7%. *Borrelia bissettii* and *Borrelia kurtenbachii* (in mixed infection with *B. burgdorferi* s.s., *B. garinii* and *B. valaisiana*) were detected in one tick from Malacky and in one tick from Rozhanovce, respectively (Hanincová et al., 2003a; Kazimírová et al., 2023). The proportions of eleven types of detected co-infections is shown in **Figure 2**.

### 3.5 Changes in the infection prevalence and diversity of *B. burgdorferi* s.l. species during the monitored years

#### 3.5.1 Železná studnička (suburban habitat, six years monitored)

**Figure 3:**
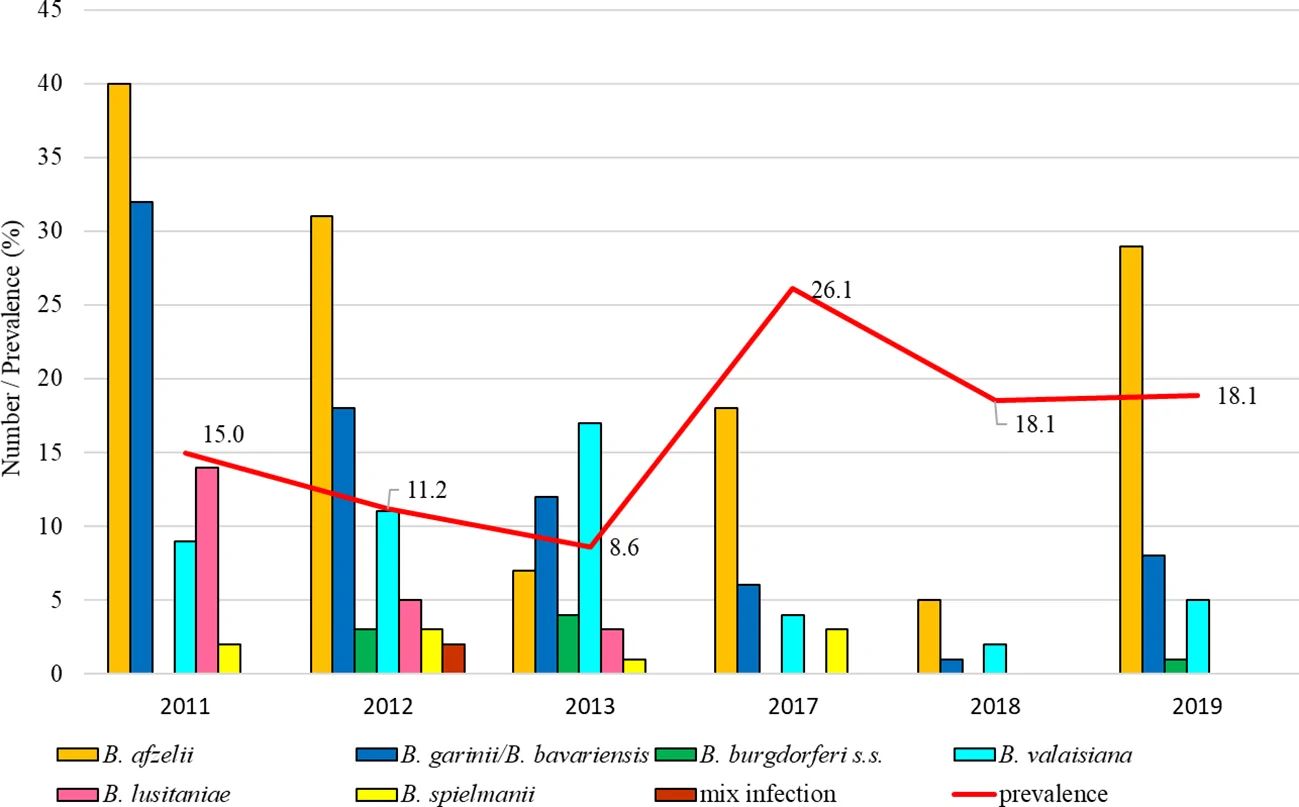
Total infection prevalence and representation of *B. burgdorferi* s.l. species in Bratislava - Železná studnička.

The total infection prevalence was 13.5% (95% CI: 12.2-15.0). The lowest prevalence was recorded in 2013 (8.1%, 95% CI: 6.5-11.3) and in 2017 tick positivity increased (26.1%, 95% CI: 19.0-34.6). Differences in the prevalence of infected ticks were significant between study years (t = 6.26, p < 0.05). *Borrelia afzelii* predominated in all studied years except for 2013 when *B. valaisiana* and *B. garinii*/*B. bavariensis* dominated. *Borrelia lusitaniae* was recorded during three study years (2011, 2012, 2013), *B. burgdorferi* s.s. in 2012, 2013 and 2019 (**Figure 3**).

#### 3.5.2 Malacky (urban park, nine years monitored)

**Figure 4:**
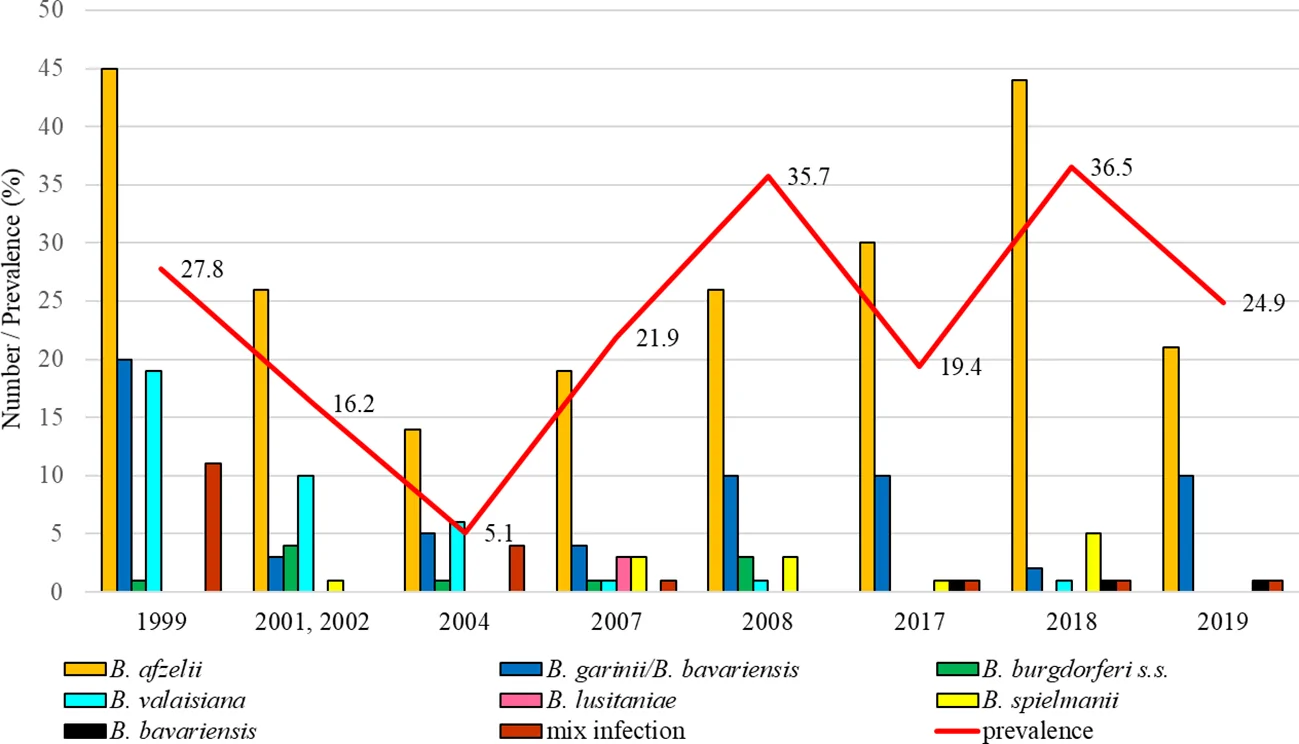
Total infection prevalence and representation of *B. burgdorferi* s.l. species in Malacky.

The total infection prevalence was 19.3% (95% CI: 17.6 – 21.0), whereas differences in the prevalence of infected ticks were significant between study years (t = 6.41, p < 0.05). The lowest prevalence of *Borrelia*-positive ticks was recorded in 2004 (5.1%, 95% CI: 3.6-7.1). *Borrelia afzelii* was dominant in all study years. *Borrelia garinii*/*B. bavariensis* was the second most frequent species during the years 1999, 2008, 2017, and 2019. *Borrelia valaisiana* had a higher representation only in 2001-2002 and 2004. *Borrelia lusitaniae* was detected only in samples collected in 2007. *Borrelia burgdorferi* s.s. and *B. spielmanii* were detected in a low percentage in several years. At this site, *B. bavariensis* was confirmed in three ticks (2017, 2018, 2019) (**Figure 4**).

#### 3.5.3 Martinské hole (natural habitat, 11 years monitored)

**Figure 5:**
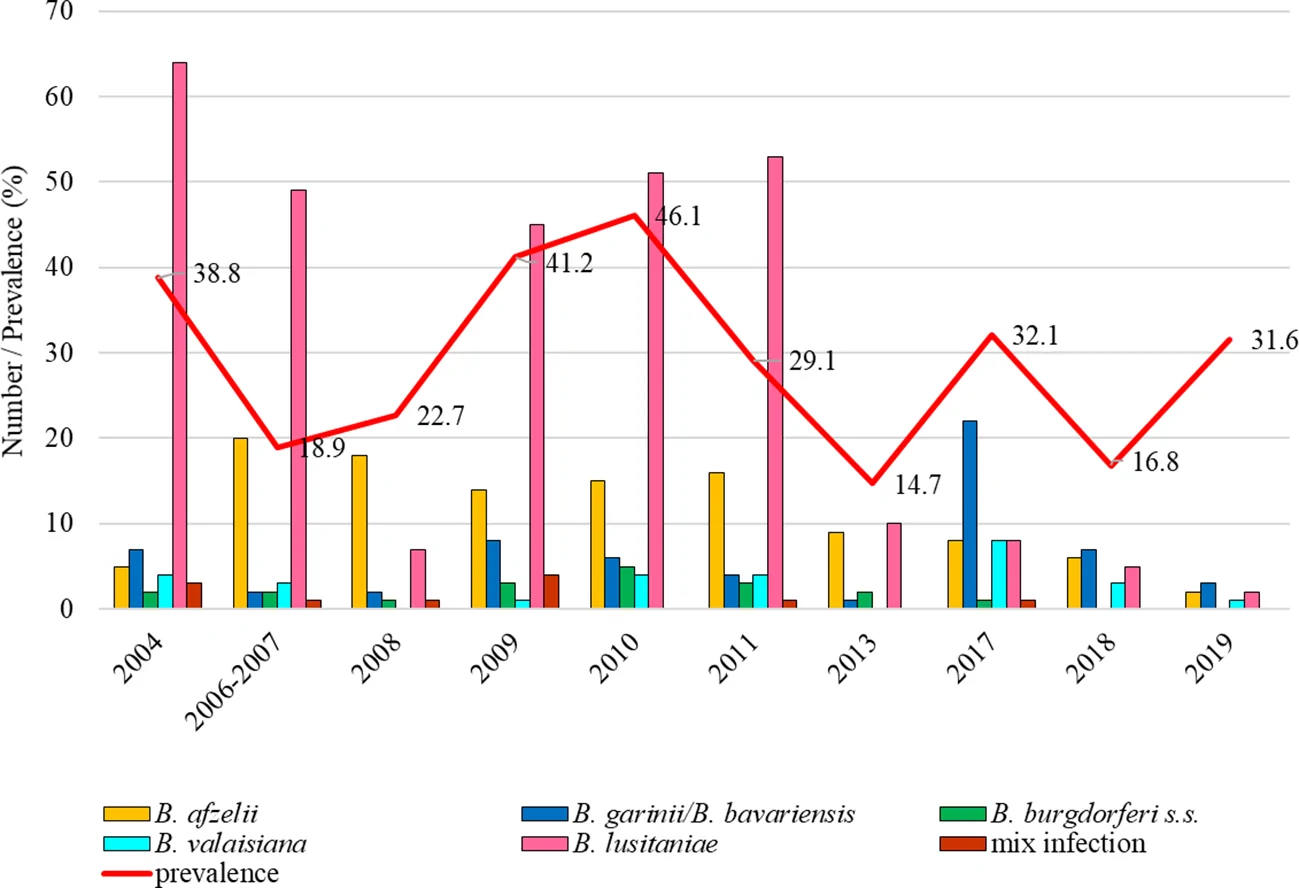
Total infection prevalence and representation of *B. burgdorferi* s.l. species in Martinské hole.

Prevalence of infected ticks fluctuated between study years (14.7%, 95% CI: 9.9-21.2; 46.1%, 95% CI: 38.9-53.4). The total infection prevalence was 28.5% (95% CI: 26.6-30.6). Differences in the prevalence of infected ticks were significant between study years (t = 8.56, p < 0.05). *Borrelia lusitaniae* clearly dominated in six years (2004, 2006-2007, 2009, 2010-2011). From 2013 the proportion of ticks infected with this species gradually decreased. *Borrelia burgdorferi* s.s. was detected in a small number of ticks during the monitored period with the exception of the last two years. *Borrelia garinii*/*B. bavariensis* predominated in 2017-2019. *Borrelia valaisiana* was recorded in a low percentage during all study years with the exception of 2008 (**Figure 5**).

#### 3.5.4 Drienovská mokraď (natural habitat, five years monitored)

**Figure 6:**
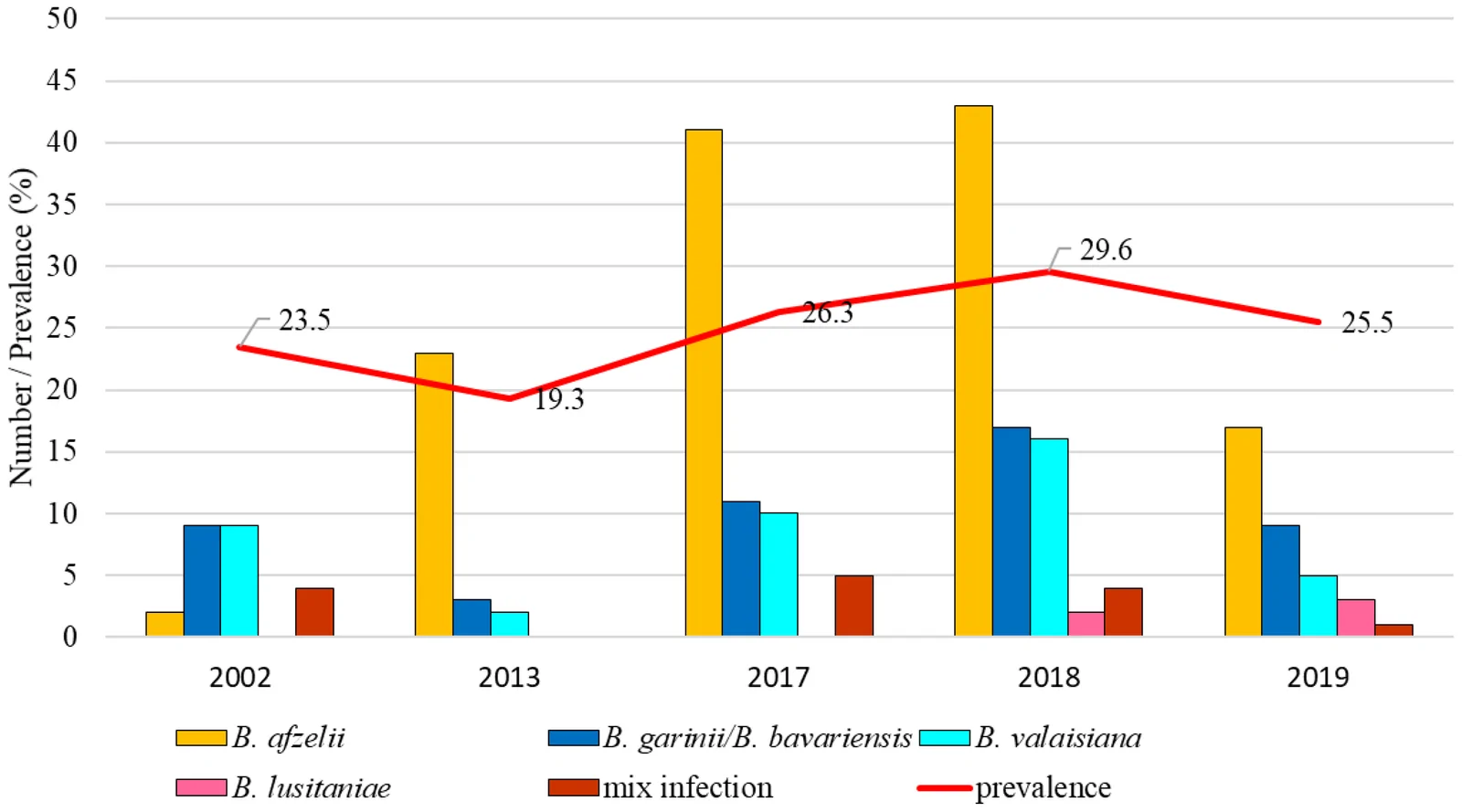
Total infection prevalence and representation of *B. burgdorferi* s.l. species in Drienovská mokraď.

The total infection prevalence was 25.8% (95% CI: 23.0-28.7). Differences in the prevalence of infected ticks were not significant between the study years (t = 14.63, p < 0.05). *Borrelia afzelii* was the dominant species in four years (2013, 2017-2019), followed by *B. garinii*/*B. bavariensis* and *B. valaisiana* which were dominant in 2002. *Borrelia lusitaniae* was found only in 2019 (**Figure 6**).

#### 3.5.5 Rozhanovce (agricultural habitat, six years monitored)

**Figure 7:**
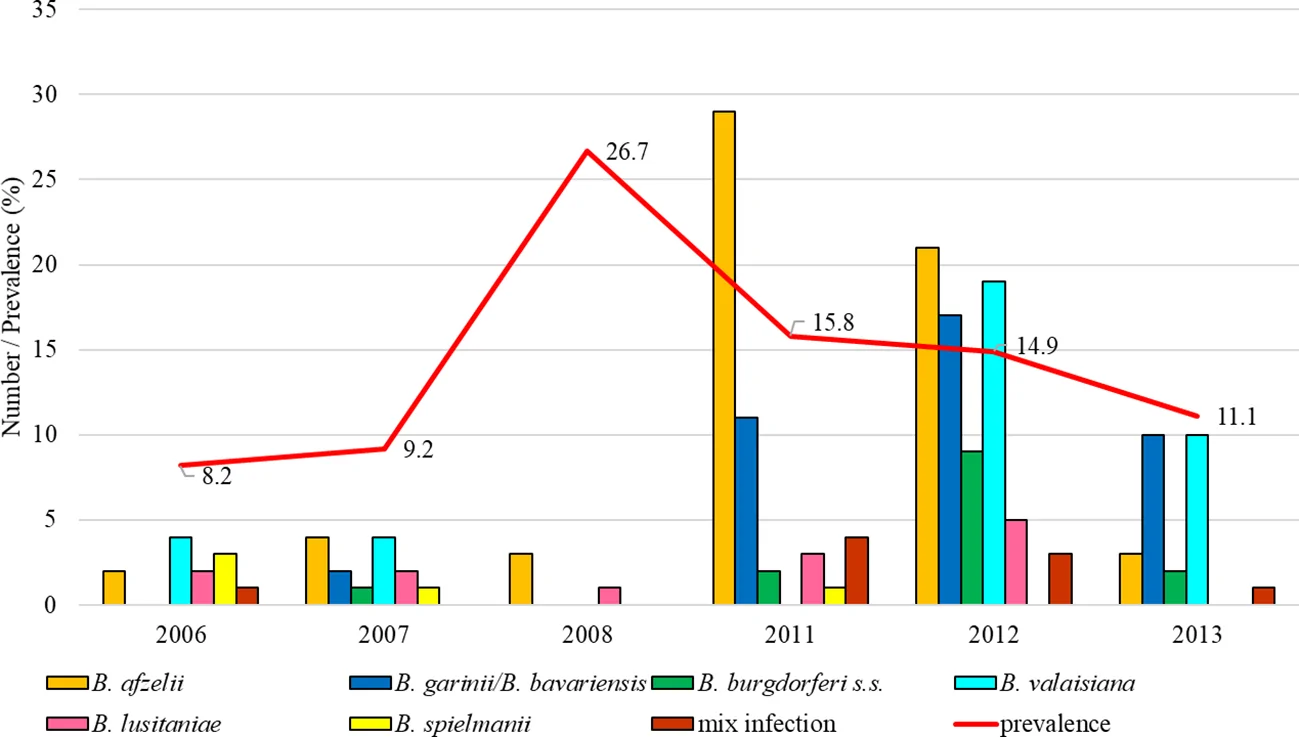
Total infection prevalence and representation of *B. burgdorferi* s.l. species in Rozhanovce.

The total infection prevalence was 13.4% (95% CI: 11.7-15.3). Differences in the prevalence of infected ticks were significant between study years (t = 5.17, p < 0.05). The most frequent species were *B. afzelii* (2007, 2008, 2011, 2012), *B. garinii*/*B. bavariensis* (predominant in 2013) and *B. valaisiana* (2007). *Borrelia burgdorferi* s.s. was detected in 2007, 2011, 2012 (with evident increase) and 2013 (**Figure 7**).

#### 3.5.6 Košice (suburban habitat, nine years monitored)

**Figure 8:**
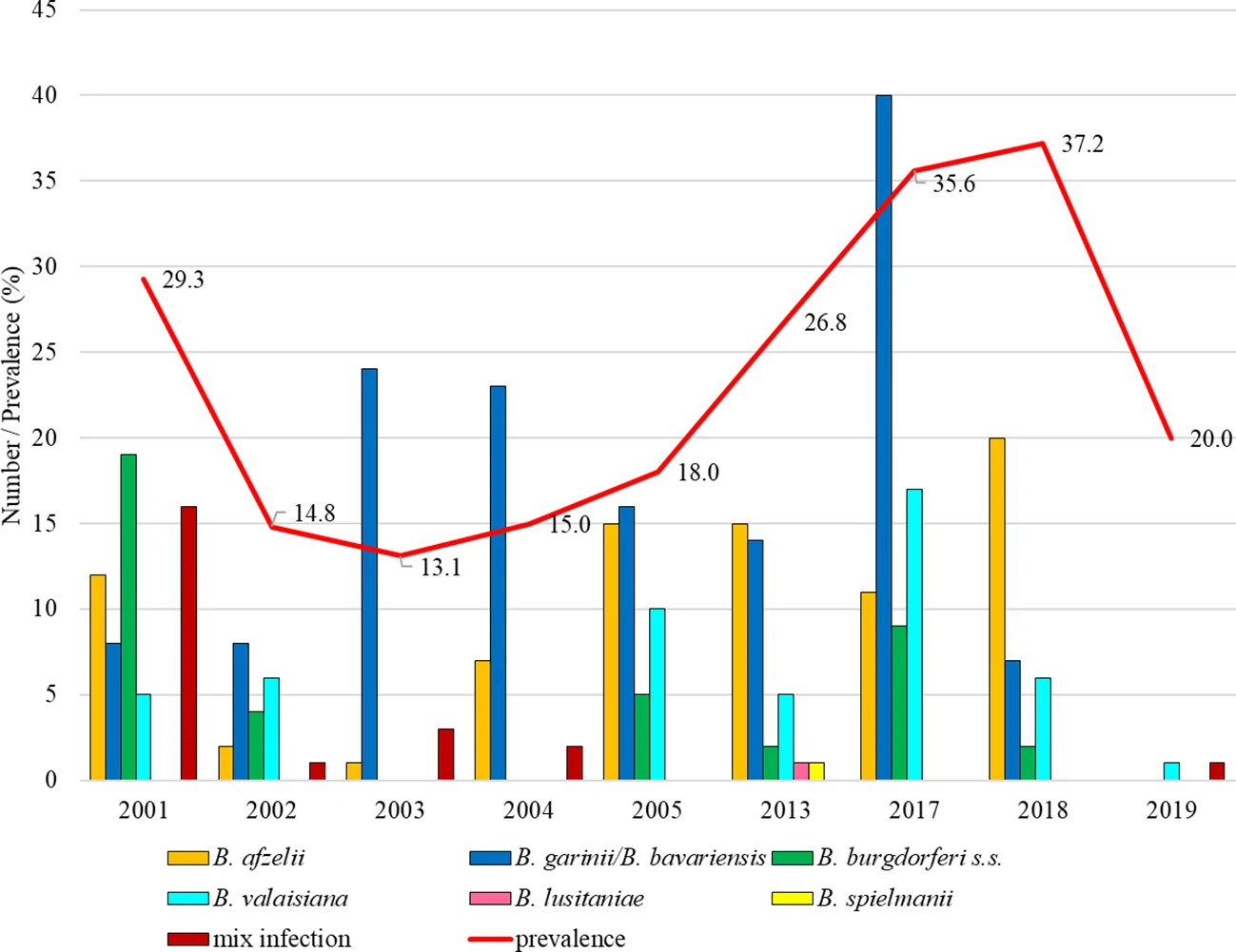
Total infection prevalence an representation of *B. burgdorferi* s.l. species in Košice.

Total infection prevalence was 22.4% (95% CI: 20.4-24.5). Differences in the prevalence of infected ticks were significant between study years (t = 7.51, p < 0.05). The lowest proportion of *Borrelia*-positive ticks was recorded in 2003 (13.1%, 95% CI: 9.3-18.3). From this year the infection prevalence was increasing up to 37.2% (95% CI: 28.8-46.4) in 2018. In the first studied year *B. burgdorferi* s.s. predominated (31.7%) and *B. garinii*/*B. bavariensis* was the most frequent in 2003, 2004, 2005 and 2017. *Borrelia afzelii* dominated in 2013 and 2018. *Borrelia valaisiana* was recorded in all years with the exception of 2003 and 2004 (**Figure 8**).

### 3.6 Prevalence of *Borrelia burgdorferi* s.l. in ticks from four habitat types

The total prevalence differed significantly among four habitat types (**Table 1**) (t = 9.66, p < 0.05). The highest prevalence was confirmed in the natural habitat (22.0%), the lowest in the urban habitat (13.2%).

**Figure 9:**
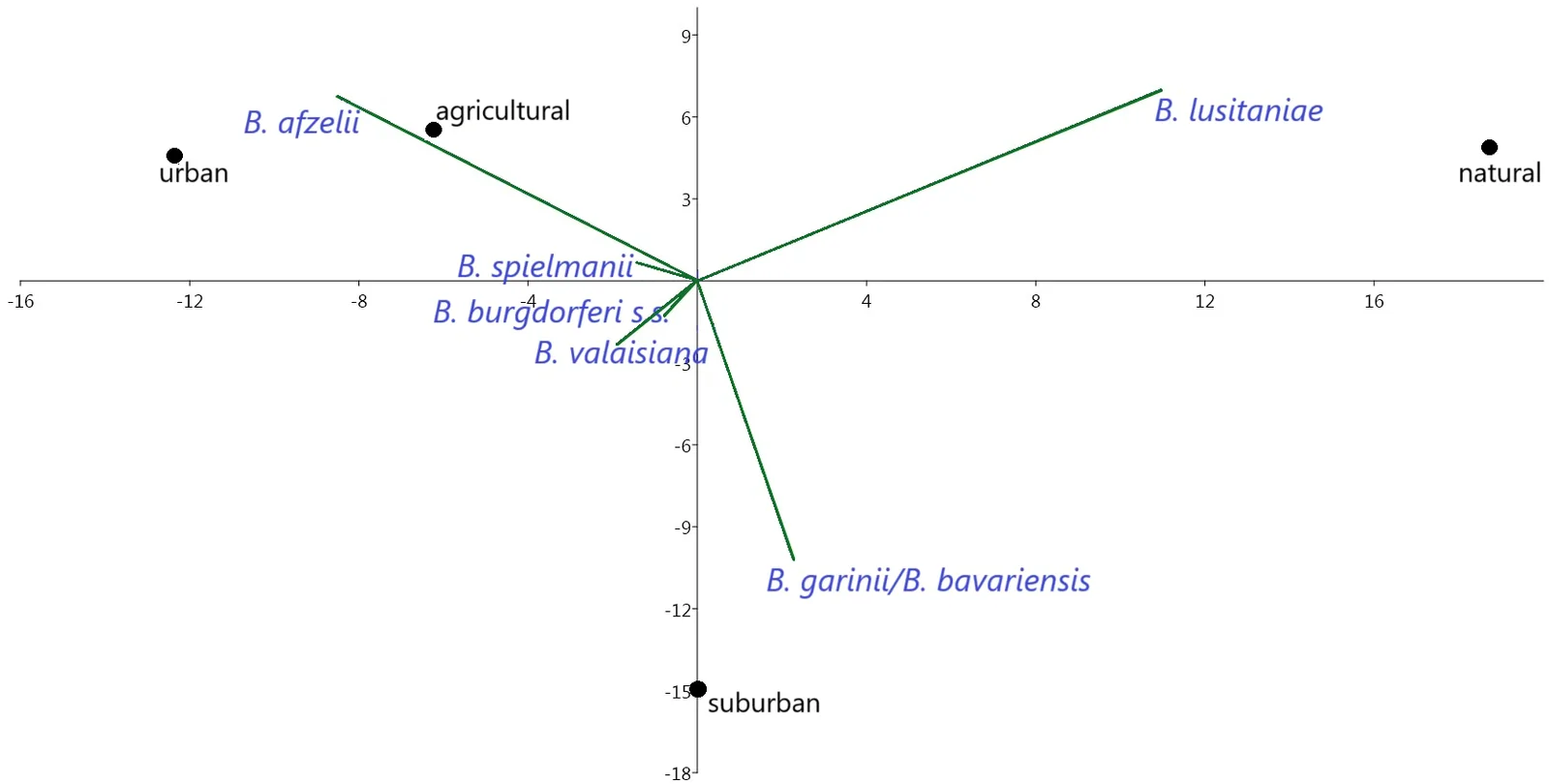
Correlations between prevalence of *B. burgdorferi* s.l. species and habitat types.

*Borrelia afzelii* predominated in all habitats, but had the highest affinity to urban and agricultural habitats. *Borrelia valaisiana* and *B. garinii*/*B. bavariensis* were the most frequent species (33.0%) at suburban sites, which was also confirmed by PCA analysis (**Table 4**; **Figure 9**). *Borrelia lusitaniae* was positively associated with the natural habitat. However, it is not possible to draw definitive conclusions based on the PCA analysis in case of *B. burgdorferi* s.s. and *B. spielmanii*. To confirm the correlation, it is necessary to obtain more samples of both agents.

**Table 4:** Number of positive *Ixodes ricinus* ticks and prevalence of *B. burgdorferi* s.l. species in urban/suburban/natural/agricultural habitats.

Habitat	No. of positive/tested ticks (%)	*B. afzelii* (%)	*B. garinii/* *B. bavariensis* (%)	*B. valaisiana* (%)	*B. lusitaniae* (%)	*B. spielmanii* (%)	*B. burgdorferi* s.s. (%)
Urban	582/4404 (13.2)	283 (48.6)	113 (19.4)	71 (12.2)	17 (2.9)	28 (4.8)	24 (4.1)
Suburban	975/5019 (19.4)	298 (30.6)	322 (33.0)	177 (18.2)	27 (2.8)	11 (1.1)	54 (5.5)
Natural	1175/5330 (22.0)	343 (29.2)	266 (22.6)	132 (11.2)	315 (26.8)	7 (0.6)	31 (2.6)
Agricultural	516/2496 (20.7)	219 (42.4)	78 (15.1)	106 (20.5)	40 (7.8)	5 (1.0)	22 (4.3)

## 4 Discussion

### 4.1 Ixodes ricinus/Ixodes inopinatus

Some studies indicate the presence of *I. inopinatus* ticks not only in dry Mediterranean regions, but also at sites in Central Europe (CE) (Chitimia-Dobler et al., 2018; Toma et al., 2021). Its occurrence was recorded in several regions of Germany (Chitimia-Dobler et al., 2017; Hauck et al., 2019), sympatric occurrence with *I. ricinus* in Romania, in western Austria and southeastern Germany (Chitimia-Dobler et al., 2018). Natural hosts include foxes, sheep and lizards (Estrada-Peña et al., 2014; Chitimia-Dobler et al., 2018). The species was recently recorded on migratory birds (Toma et al., 2021). On the other hand, the situation with regards to the presence of this tick species in CE is complicated (Kahl and Gray, 2023) and some studies suggest that it does not occur in CE (Rollins et al., 2023). Published data about *I. inopinatus* described by Estrada-Peña et al. (2014) raise the question of whether this species also occurs in Slovakia. Based on published data, we sequenced randomly selected samples of ticks that were morphologically determined as *I. ricinus*. Our preliminary analyses have not confirmed the presence of *I. inopinatus* in Slovakia.

### 4.2 *Borrelia burgdorferi* s.l.

*Borrelia burgdorferi* s.l. complex is the subject of many epidemiological studies in Europe (Rauter and Hartung, 2005; Strnad et al., 2017; Estrada-Peña et al., 2018). Global warming and climate change in Central Europe (CE) has an impact on the geographic distribution and seasonal activity of ticks and pathogens they carry as well as on the occurrence and population density of their hosts (Lindgren et al., 2000; Nuttall, 2022).

Long-term studies on the prevalence of *B. burgdorferi* s.l. are rare although monitoring of changes in the prevalence and species diversity at the same sites can provide precious information on the impact of global changes on ticks and tick-borne pathogens. In the present study, we summarised data on the infection prevalence and distribution of species of the *B. burgdorferi* s.l. complex in questing *I. ricinus* ticks from 16 study sites in Slovakia and from over 20 years (1999-2019). The large package of data shows considerable spatial variability within the *B. burgdorferi* s.l. complex even in such a small geographic area as Slovakia. In addition, marked temporal differences in the prevalence of infected ticks were observed at the same localities. These results are consistent with other European studies on spatial and temporal changes in *Borrelia* prevalence (e.g. Hönig et al., 2015; Okeyo et al., 2020; Hartemink et al., 2021; Medlock et al., 2022; Glass et al., 2023).

The abundance of *I. ricinus* is influenced by many factors and varies depending on the month/time of collection, as well as on changes that occur at individual sites over the years (for example, clearing of dense undergrowth in city parks, mowing of grassy areas etc.). Usually, nymphs are spread more uniformly than adult ticks and larvae (Gray et al., 2021). The relative abundance of questing *I. ricinus* nymphs in a three-year study from five selected central European countries was found to be associated with climatic conditions, but mainly with the Normalized Difference Vegetation Index, and did not significantly depend on land use categories (Rosà et al., 2018). In Slovakia, the highest density of nymphs was found in green urban sites, whereas by summarizing data from all studied countries and habitat types (urban, suburban, natural, agricultural), density of nymphs was highest in the natural habitats (Rosà et al., 2018). In our analysed datasets, nymphs were more abundant at ten study sites whereas adult ticks prevailed at five study sites. We recorded a higher overall infection prevalence of *B. burgdorferi* s.l. (18.8%) compared to the meta-analyses for Central Europe: 13.7% (Rauter and Hartung, 2005) and 12.3% (Strnad et al., 2017). The prevalence of *Borrelia*-infected nymphs (15.1%) in Slovakia was higher than 10.1% and 11.8% in the datasets from 1984-2003 (Rauter and Hartung, 2005) and from 2010-2016 (Strnad et al., 2017), respectively. The situation was similar for adult ticks with 24.3% positive ticks in 1999-2019 (our study) in contrast to previous studies - 18.6% (Rauter and Hartung, 2005) and 14.9% (Strnad et al., 2017). In contrast, the overall *Borrelia* prevalence in nymphs based on data from Slovakia, Germany and Italy for 2011-2013 was 19.3% (Rosà et al., 2018).

In our study, the lowest infection prevalence was recorded in urban areas (13.2%) while in a long-term study from the city of Hanover (Germany) the prevalence of infected ticks was as high as 25.5% (Glass et al., 2023). In comparison with urban sites of Slovakia, higher *Borrelia* prevalence was recorded in suburban, natural and agricultural sites which have more diverse vertebrate hosts composition (Rizzoli et al., 2014; Kazimírová et al., 2023). Spatial and temporal changes in the borrelial prevalence even within small geographical areas were reported from Slovakia, e.g., by Pangrácová et al. (2013) and Kazimírová et al. (2023). Depending on the location and habitat type, prevalence of infected ticks ranged from 1.0% to 27.8% (nymphs) and from 0% to 49.4% (adults). Total prevalence was found to range between 0% and 38.3% in urban/suburban sites, between 8.0% and 29.4% in natural sites and between 32.3% and 38.1% in agricultural sites and ecotones (see Kazimírová et al., 2023).

The distribution of *B. burgdorferi* s.l. species in a site depends on the presence of appropriate reservoir hosts (Wolcott et al., 2021; Steinbrink et al., 2022; Estrada-Peña et al., 2024). In total, nine species were identified in our study. *Borrelia afzelii*, *B. garinii*/*bavariensis* and *B. valaisiana* were the most frequent and were confirmed at all study sites. These results are consistent with data from other European studies (Strnad et al., 2017; Mysterud et al., 2019). In the suburban habitat, bird-associated species (*B. garinii* and *B. valaisiana*) prevailed, which may be related to the high number of songbirds in urban forest parks. *Borrelia lusitaniae* was confirmed in 12.8% positive samples from Slovakia, which is a higher proportion than 7% in the study of Strnad et al. (2017). This was caused by a high proportion of *B. lusitaniae*-positive ticks (73.7%) from a natural focus in a mountain region in Slovakia, Martinské hole Mts., that is not typical of Central Europe (Rusňáková Tarageľová et al., 2016). According to our results, this species has an affinity to the natural habitat, but the results are skewed by high number of *B. lusitaniae*-positive ticks from one study site only. By our previous methodology, identification of *B. bavariensis* was not possible, but we assume that confirmed infections with *B. garinii* might have included this species. The presence of *B. bavariensis* was confirmed in Slovakia by Hamšíková et al. (2017) and in a former study as the *B. garinii* ospA serotype 4 (Lenčáková et al., 2006). The rare species *B. bissettii* and *B. kurtenbachii* have been detected in Slovakia only once, and in coinfections with other *Borrelia* species (Hanincová et al., 2003b; Kazimírová et al., 2023).

Global changes are affecting all components of the natural foci of tick-borne diseases (Gray et al., 2009). Changes in the diversity of *Borrelia* species at the monitored sites in Slovakia were probably due to the influence of several factors:

- spectrum and/or abundance of competent reservoir hosts for *B. burgdorferi* s.l.;
- changes in the environment (natural and/or due to human activities);
- various methodologies for the detection of *B. burgdorferi* s.l.;
- unequal numbers of collected ticks in individual years and study sites.

As *B. afzelii* is associated with rodents, increasing/decreasing of the prevalence of this species may be driven by natural fluctuations in rodent population densities (Glass et al., 2023) as well as by changes in the species composition of the rodent communities. For example, high population densities of the common vole (*Microtus arvalis*), the major reservoir host for *B. afzelii* (Radzijevskaja et al., 2013), were registered Europe-wide (Jacob et al., 2020; Heroldová et al., 2021), and the striped field mouse (*Apodemus agrarius*) is currently expanding its range (Tulis et al., 2023). In years with low population densities of rodents, song birds being reservoirs of *B. garinii* and *B. valaisiana* may become the major feeding hosts and sources of infection for *I. ricinus*, especially in urban and suburban areas (Rizzoli et al., 2014). *Borrelia lusitaniae* dominated at Martinské hole until 2013, but since then its prevalence has been decreasing. At this location, a large area of the forest was destroyed, so the composition of tick hosts has probably changed.

## 5 Conclusions

The analysis of a large dataset of *B. burgdorferi* s.l. infections in questing *I. ricinus* nymphs and adults collected during over 20 years from a number of sites representing different habitat types of Slovakia revealed (1) the presence of infected ticks in all explored sites, (2) spatial and temporal differences in the prevalence of infections and in the diversity of *Borrelia* species. Nine species were found to occur, with *B. afzelii* and *B. garinii* prevailing. Presence of human-pathogenic *Borrelia* species was confirmed in all studied sites. Depending on the site, these comprised *B. afzelii* and *B. garinii, B. burgdorferi* s.s, *B. bavariensis* and *B. spielmanii.* Urban/suburban green areas and parks have been identified as foci for circulation of pathogenic *Borrelia* species and represent areas with high epidemiological risk. Long-term monitoring of the presence and prevalence of *Borrelia* infections in ticks is needed to unravel new foci of the pathogens and confirm the maintenance of existing foci in a changing environment.

## Data Availability

The original contributions presented in the study are included in the article/**Supplementary Material**. Further data are publicly available and can be found here: https://doi.org/10.6084/m9.figshare.27967431.v1. Further inquiries can be directed to the corresponding author.
