# Supplementary material for: Two decades of research on Borrelia burgdorferi sensu lato in questing Ixodes ricinus ticks in Slovakia
Source: Front Cell Infect Microbiol. 2024 Dec 13;14:1496925. doi: 10.3389/fcimb.2024.1496925 (PMC11673768; doi:10.3389/fcimb.2024.1496925)

Supplementary Figure 1. Representation of *B. burgdorferi* s.l. species in *Ixodes ricinus* at all study sites. BA-PB: Bratislava - Podunajské Biskupice; BA-SAS: Bratislava – Slovak Academy of Sciences; BA-HP: Bratislava – Horský park; BA-ŽS: Bratislava – Železná studnička

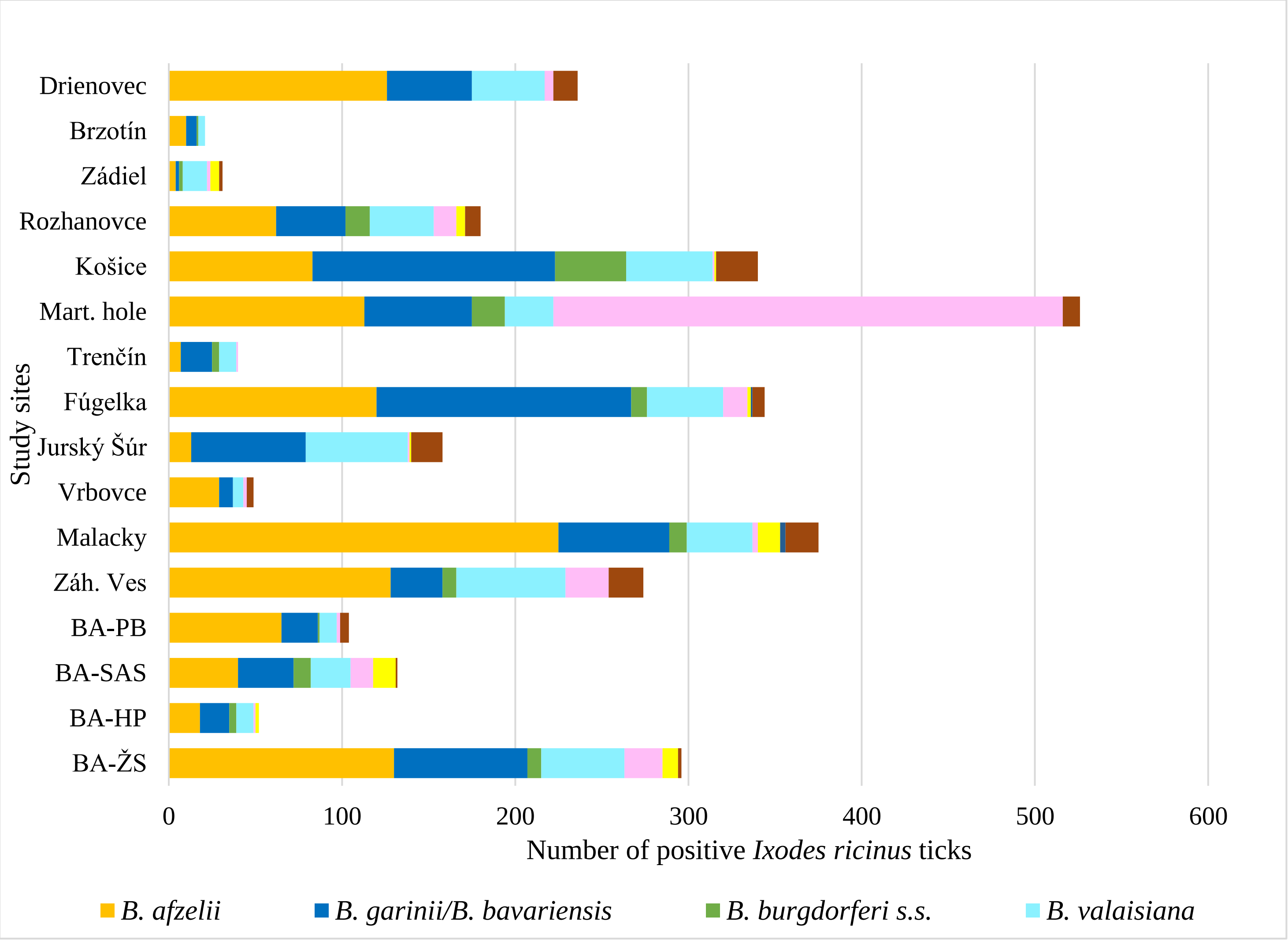

Supplement: Supplementary file 1 [file DataSheet1.pdf]
