# Supplementary material for: Two decades of research on Borrelia burgdorferi sensu lato in questing Ixodes ricinus ticks in Slovakia
Source: Front Cell Infect Microbiol. 2024 Dec 13;14:1496925. doi: 10.3389/fcimb.2024.1496925 (PMC11673768; doi:10.3389/fcimb.2024.1496925)
Supplement: Supplementary file 2 [file Supplementaryfile1.pdf]

Supplementary Table 1. Study sites and years of tick collections

Locality	Years of tick collections	District	Region	Altitude	Geographical coordinates	Mountain and valleys range	Habitat type	References
Horský park	2011, 2012	Bratislava	Bratislava	220 m a. s. l.	48°9'24"N, 17°5'41"E	Small Carpathians Mts.	urban - sylvatic park in the middle of the town	Vaculová et al. 2019; Kazimírová et al. 2023
Slovak Academy of Sciences	2011-2013	Bratislava	Bratislava	195 m a. s. l.	48° 9′ 46.37″ N, 17° 4′ 29.64″ E	Small Carpathians Mts.	urban - area with park where are grassy areas and deciduous forested areas	Chvostáč et al. 2018; Kazimírová et al. 2023
Malacky	1999, 2001, 2002, 2004, 2007, 2008, 2017-2019	Malacky	Bratislava	170 m a. s. l.	48° 26′ 23.18″ N, 17° 1′ 52.55″ E	Small Carpathians Mts.	urban - sylvatic park in the middle of the town	Hanincová et al. 2003a; Tarageľová et al. 2005; Tarageľová et al. 2008
Železná Studnička	2011-2013, 2017-2019	Bratislava	Bratislava	185 m a. s. l.	48°11′8″N, 17°4′38″E	Small Carpathians Mts.	suburban - deciduous forest park, recreational area	Vaculová et al. 2019; Kazimírová et al. 2023
Podunajské Biskupice	1999	Bratislava	Bratislava	140 m a. s. l.	48°7′ 47.6″ N, 17°12′ 39″ E	Small Carpathians Mts.	suburban - deciduous forest situated near the river Danube	Hanincová et al. 2003a
Jurský Šúr (The Šúr Nature reserve)	2001, 2002, 2013	Pezinok	Bratislava	146 m a. s. l.	48° 14′ 18″ N, 17° 13′ 41″ E	interface of the Podunajská nížina Plain and Small Carpathians Mts.	suburban - alder forest with a transition to a floodplain forest	Hanincová et al. 2003a; Tarageľová et al. 2005
Košice	2001-2005, 2013, 2017-2019	Košice	Košice	273 m a. s. l.	48° 44′ 18″ N, 21° 16′ 57″ E	Košice basin	suburban - deciduous forest located in the eastern part of town	Derdáková 2004
Trenčín	2010	Trenčín	Trenčín	325 m a. s. l.	48° 53′ 28.78″ N, 18° 2′ 50.2″ E	Strážov Mts.	suburban - sylvatic park in the middle of the town	Unpublished
Fúgelka	2011-2013	Bratislava	Bratislava	394 m a. s. l.	48° 24′ 51.35″ N, 17° 18′ 5.25″ E	Small Carpathians Mts.	natural - non-fragmented deciduos forest	Kazimírová et al. 2016; Svitálková et al. 2015
Martinské hole Mountains	2004, 2006-2011, 2013, 2017-2019	Martin	Žilina	630-1070m a. s. l.	49° 5′ 38.4″ N, 18° 50′ 2.4″ E	Malá Fatra, Turčianska kotlina basin	natural - mountainous mixed forest and fir-pine and pine forests at higher altitude	Rusňáková Tarageľová et al. 2016
Drienovec	2002, 2013, 2017-2019	Košice	Košice area	190 m a. s. l.	48° 38′ 20″ N, 20° 40′ 44″ E	interface of Košice basin and Slovenský kras National Park (Slovak Karst)	natural - xerothermic habitats, dry oak forests, reed habitats alternating with sedge and willows, ruderals, meadows	Tarageľová et al. 2005; Tarageľová et al. 2008; Mtierová et al. 2020
Zádiel	2006, 2007	Košice	Košice area	300 m a. s. l.	48° 36′ 52.92″ N, 20° 49′ 58.44″ E	Slovenský kras Slovak National Park (Slovak Karst)	natural - xerothermic dry fragmented forest	Haklová-Kočíková et al. 2014
Brzotín	2002	Rožňava	Košice area	290 m a. s. l.	48° 37′ 49″ N, 20° 29′ 52″ E	interface of Košice basin and Slovenský kras National Park (Slovak Karst)	natural - xerothermic dry fragmented forest	Tarageľová et al. 2008
Záhorská Ves	1999, 2000, 2004	Malacky	Bratislava	162 m a. s. l.	48° 22′ 50.78″ N, 16° 50′ 41.69″ E	Small Carpathians Mts.	agricultural - interface of forest and farmland on the edge of the village	Hanincová 2003a, b
Vrbovce	2017-2019	Myjava	Trenčín	409 m a. s. l.	48° 47′ 56.76″ N, 17° 28′ 7.32″ E	foothills of Biele Karpaty Mts.	agricultural - organic farm with meadows and pastures combined with arable land and various wooded parts of ravines and ditches	Unpublished

Rozhanovce	2006-2008, 2011-2013	Košice	Košice	260 m a. s. l.	48° 45′ 0″ N, 21° 21′ 0″ E	Košice basin	agricultural - game reserve at agricultural site with fragmented habitats comprising patches of oak-hornbeam forest and a few cultivated fields and meadows	Špitalská et al. 2016; Kazimírová et al. 2023

References

Derdáková, M. (2004). Molekulárna epidemiológia lymskej boreliózy. [Dissertation thesis]. [Košice]: Institute of Parasitology SAS.

Haklová-Kočíková, B., Hižňanová, A., Majláth, I., Račka, K., Harris, D.J., Földvári, G., et al. (2014). Morphological and molecular characterization of *Karyolysus* - a neglected but common parasite infecting some European lizards. *Parasit. Vectors.* 7, 555. doi: 10.1186/s13071-014-0555-x

Hanincová, K., Schäfer, S. M., Etti, S., Sewell, H. S., Tarageľová, V., Žiak, D., et al. (2003a). Association of *Borrelia afzelii* with rodents in Europe. *Parasitology* 126, 11-20. doi: 10.1017/s0031182002002548

Hanincová, K., Tarageľová, V., Koči, J., Schäfer, S. M., Hails, R., Ullmann, A. J., et al. (2003b). Association of *Borrelia garinii* and *B. valaisiana* with songbirds in Slovakia. *Appl. Environ. Microbiol.* 69 (5), 2825-2830. doi: 10.1128/AEM.69.5.2825-2830.2003

Chvostáč, M., Špitalská, E., Václav, R., Vaculová, T., Minichová, L., Derdáková, M. (2018). Seasonal patterns in the prevalence and diversity of tick-borne *Borrelia burgdorferi* sensu lato, *Anaplasma phagocytophilum* and *Rickettsia* spp. in an urban temperate forest in South Western Slovakia. *Int. J. Environ. Res. Public Health.* 15 (5), 994. doi: 10.3390/ijerph15050994

Kazimírová, M., Mahríková, L., Hamšíková, Z., Stanko, M., Golovchenko, M., Rudenko, N. (2023). Spatial and temporal variability in prevalence rates of members of the *Borrelia burgdorferi* species complex in *Ixodes ricinus* ticks in urban, agricultural and sylvatic habitats in Slovakia. *Microorganisms.* 11 (7), 1666. doi: 10.3390/microorganisms11071666

Mtierová, Z., Derdáková, M., Chvostáč, M., Didyk, Y. M., Mangová, B., Rusňáková Tarageľová, V., et al. (2020). Local population structure and seasonal variability of *Borrelia garinii* genotypes in *Ixodes ricinus* ticks, Slovakia. *Int. J. Environ. Res. Public Health*. 17 (10), 3607. doi: 10.3390/ijerph17103607

Svitálková, Z., Haruštiaková, D., Mahríková, L., Berthová, L., Slovák, M., Kocianová, E., Kazimírová, M. (2015). *Anaplasma phagocytophilum* prevalence in ticks and rodents in an urban and natural habitat in South-Western Slovakia. *Parasit. Vectors* 8, 276. doi: 10.1186/s13071-015-0880-8

Špitalská, E., Stanko, M., Mošanský, L., Kraljik, J., Miklisová, D., Mahríková, L., et al. (2016). Seasonal analysis of *Rickettsia* species in ticks in an agricultural site of Slovakia. *Exp. Appl. Acarol.* 68, 315-324. doi: 10.1007/s10493-015-9941-0

Rusňáková Tarageľová, V., Mahríková, L., Selyemová, D., Václav, R., Derdáková, M. (2016) Natural foci of *Borrelia lusitaniae* in a mountain region of Central Europe. *Ticks Tick Borne Dis.* 7, 350-356. doi: 10.1016/j.ttbdis.2015.12.006

Tarageľová, V., Koči, J., Hanincová, K., Olekšák, M., Labuda, M. (2005). Songbirds as hosts for ticks (Acari, Ixodidae) in Slovakia. *Biologia.* 60, 529-537.

Taragel'ová, V., Koči, J., Hanincová, K., Kurtenbach, K., Derdáková, M., Ogden, N.H., et al. (2008). Blackbirds and song thrushes constitute a key reservoir of *Borrelia garinii*, the causative agent of borreliosis in Central Europe. *Appl. Environ. Microbiol.* 74, 1289-1293. doi: 10.1128/AEM.01060-07

Vaculová, T., Derdáková, M., Špitalská, E., Václav, R., Chvostáč, M., Rusňáková Tarageľová, V. (2019). Simultaneous occurrence of *Borrelia miyamotoi*, *Borrelia burgdorferi* sensu lato, *Anaplasma phagocytophilum* and *Rickettsia helvetica* in *Ixodes ricinus* ticks in urban foci in Bratislava, Slovakia. *Acta Parasitol.* 64, 19-30. doi: 10.2478/s11686-018-00004-w
