# Supplementary material for: Two decades of research on Borrelia burgdorferi sensu lato in questing Ixodes ricinus ticks in Slovakia
Source: Front Cell Infect Microbiol. 2024 Dec 13;14:1496925. doi: 10.3389/fcimb.2024.1496925 (PMC11673768; doi:10.3389/fcimb.2024.1496925)
Supplement: Supplementary file 3 [file Supplementaryfile2.pdf]

Supplementary Table 2. Sequences of primers and probes used in PCRs for detection of *B. burgdorferi* s.l.

Gene	Primers	Oligonucleotide sequence (5′-3′)	Reference	Species detection
rrfA-rrlB intergenic spacer	IGSa	CGA CCT TCT TCG CCT TAA AGC	Derdáková et al. 2003	RFLP analysis/sequencing
	IGSb	AGC TCT TAT TCG CTG ATG GTA		
Flagellin	Cf	GCA GTT CAA TCA GGT AAC GG	Fukunaga et al. 1996	
	Dr	AGG TTT TCA ATA GCA TAC TC		
rrf (5S)–rrl (23S) intergenic spacer	23SN1	ACCATAGACTCTTATTACTTTGAC	Rijpkema et al. 1995	reverse line blot/sequencing
	23SC1	TAAGCTGACTAATACTAATTACCC		
	23SN2	ACCATAGACTCTTATTACTTTGACCA		
	5SCB	biotin-GAGAGTAGGTTATTGCCAGGG		
23S rRNA	Bb23Sf	CGAGTCTTAAAAGGGCGATTTAGT	Courtney et al. 2004	sequencing
	Bb23Sr	GCTTCAGCCTGGCCATAAATAG		
	TaqMan probe Bb23Sp-FAM	AGATGTGGTAGACCCGAAGCCGAGTG		

References

Courtney, J.W., Kostelnik, L.M., Zeidner, N.S., Massung R.F. (2004). Multiplex real-time PCR for detection of *Anaplasma phagocytophilum* and *Borrelia burgdorferi*. *J. Clin. Microbiol.* 42 (7), 3164-8. doi: 10.1128/JCM.42.7.3164-3168.2004

Derdáková, M., Beati, L., Pet'ko, B., Stanko, M., Fish, D. (2003). Genetic variability within *Borrelia burgdorferi* sensu lato genospecies established by PCR-single-strand conformation polymorphism analysis of the rrfA-rrlB intergenic spacer in *Ixodes ricinus* ticks from the Czech Republic. *Appl. Environ. Microbiol.* 69 (1), 509-516. doi: 10.1128/AEM.69.1.509-516.2003

Fukunaga, M., K. Okada, M. Nakao, T. Konishi, Y. Sato (1996). Phylogenetic analysis of *Borrelia* species based on flagellin gene sequences and its application for molecular typing of Lyme disease borreliae. *Int. J. Syst. Bacteriol.* 46**,** 898-905. doi: 10.1099/00207713-46-4-898

Rijpkema, S. G. T., Molkenboer, M. J. C. H., Schouls, L. M., Jongejan, F., Schellekens, J. F. P. (1995). Simultaneous detection and genotyping of three genomic groups of *Borrelia burgdorferi* sensu lato in Dutch *Ixodes ricinus* ticks by characterization of the amplified intergenic spacer region between 5S and 23S ribosomal RNA genes. *J. Clin. Microbiol.* 33, 3091–3095. doi: 10.1128/jcm.33.12.3091-3095.1995
